# A murine model of gestational diabetes reveals MASLD risk and alterations in markers of hepatic mitochondrial metabolism

**DOI:** 10.3389/fendo.2025.1498764

**Published:** 2025-06-25

**Authors:** Grace E. Shryack, Alexa A. Krause, Simone Hernandez Ruano, Laura C. Schulz, Kathleen A. Pennington, R. Scott Rector

**Affiliations:** ^1^ Research Service, Harry S Truman Memorial Veterans Medical Center, Columbia, MO, United States; ^2^ NextGen Precision Health, University of Missouri, Columbia, MO, United States; ^3^ Department of Nutrition and Exercise Physiology, University of Missouri, Columbia, MO, United States; ^4^ Department of Obstetrics and Gynecology, Baylor College of Medicine, Houston, TX, United States; ^5^ Deparment of Obstetrics, Gynecology, and Women’s Health, University of Missouri, Columbia, MO, United States

**Keywords:** GDM, MASLD, NAFLD, mitochondria, liver

## Abstract

**Introduction:**

Gestational Diabetes Mellitus (GDM) impacts roughly 1 in 7 pregnancies and results in metabolic dysfunction-associated steatotic liver disease (MASLD) in 30% of these women. Nonetheless, there exists a dearth of investigation into the relationship between GDM and MASLD. Here, we sought to investigate the potential role of hepatic mitochondrial function in GDM and MASLD.

**Methods:**

One week prior to conception and throughout pregnancy, mice were fed either a low-fat control diet (CD) or a high-fat, high-sucrose (HFHS) diet to induce an established model of GDM. Maternal livers were collected at day 0, 6.5, 13.5 and 17.5 of pregnancy. Hepatic markers (via mRNA and western blot analyses) of mitochondrial biogenesis, autophagy, mitophagy, activity, and function were assessed, as well as markers of inflammation and antioxidant status were evaluated.

**Results:**

Progressing gestation in both CD and GDM dams significantly decreased protein and mRNA markers of hepatic mitochondrial biogenesis (*Pgc1-α, Tfam*), autophagy (*Atg5, Sqstm1*), mitophagy (*Pink1, Bnip3*) and lipid handling (*Ampk*, pAMPK/AMPK, FAS, ACC, pACC, *Mttp*) with a main effect for time (P<0.05). HFHS-induced model of GDM lead to significant elevations in liver triglycerides and NAFLD Activity Score (NAS) (P<0.0001, P<0.0001) independent of body weight gain during gestation. MASLD development in the GDM mice occurred in conjunction with significant reductions in hepatic mitochondrial activity at day 6.5 (citrate synthase, p<0.01) and day 17.5 (β-HAD, citrate synthase, P<0.001) compared to CD mice. However, GDM lead to elevated protein and/or mRNA markers of mitochondrial biogenesis (*Tfam*), mitophagy (BNIP3, *Bnip3, Sqstm1, Pink1*), lipid handling (*Mttp*), inflammation (*Il-1β, Tnf-α, Tgf-β)* and antioxidant defense (*Gxp1, Nfe2l2, Sod2*) (P<0.05).

**Discussion:**

Pregnancy, independent of diet, decreased markers of liver mitochondrial biogenesis, autophagy, and mitophagy in dams. The GDM mouse model exhibited elevated hepatic TG and NAS, as well as decreased liver mitochondrial activity. These findings demonstrate that pregnancy and GDM significantly impact maternal liver mitochondrial metabolism and unveil new insight on the potential relationship between MASLD and GDM.

## Introduction

The diagnosis of glucose intolerance during pregnancy or Gestational Diabetes Mellitus (GDM), affects up to 14% of pregnancies globally (1, 2). Although pregnancy is a natural state of insulin resistance, women with GDM face a worsened level of insulin resistance coupled with glucose intolerance (3–8). This disease can then further be exacerbated by a lack of beta cell expansion in the pancreas, leading to reduced insulin secretion (9–11). This failure to adapt to the metabolic demands of pregnancy is associated with an increased risk of type 2 diabetes, GDM in subsequent pregnancies, hypertension, cardiovascular disease, increased body mass index, and cesarean delivery (12). Moreover, GDM induces adverse outcomes in the fetus, including increased risk for preterm birth, macrosomia, and type 2 diabetes (12).

Recently, studies have found that women with GDM have a heightened risk for developing metabolic dysfunction-associated steatotic liver disease (MASLD), formerly known as nonalcoholic fatty liver disease (NAFLD), during pregnancy and post-partum (13–15). Women with MASLD were three times more likely to develop GDM (16, 17). Furthermore, compared to their healthy, pregnant counterparts, women with GDM display alterations in hepatic serum metabolites such as lysophosphatidylcholine, glycerophospholipids, monoacylglycerol, serine, proline, leucine, and isoleucine (18, 19). These metabolites are involved in lipid and amino acid metabolism (19, 20), and such alterations of these metabolites may help explain the metabolic impact of GDM (21–23), but a mechanistic link between GDM and MASLD has yet to be determined.

MASLD, characterized by a greater than 5% of hepatocytes containing lipid, is the most common form of chronic liver disease (24, 25). MASLD is linked to several metabolic disorders including type 2 diabetes, cardiovascular disease, and obesity (24, 26). Although the specific cause of MASLD is unknown, there are a number of processes that influence this disease, such as alterations in lipid metabolism and mitochondrial function (27–30). The hepatic mitochondrion is responsible for the breakdown of different substrates via glycolysis and fatty acid oxidation which then feed into the TCA cycle and activates ATP synthesis via the electron transport chain, a process known as respiration (31–34). Further, functional mitochondria are in a constant cycle of turnover via biogenesis, autophagy, and mitophagy. During MASLD, mitochondrial respiration, metabolism, activity, and turnover are impaired (35–37). This creates a pool of poorly functioning mitochondria that exhibits inflammation, increases reactive oxygen species generation, and decreases in antioxidant capacity (35). The health of a hepatic mitochondria depends on the interaction between these processes and when impaired significantly contributes to the development and progression of MASLD (32, 37).

As stated previously, there is limited investigation into the mechanistic links between GDM and MASLD. Previous murine models of GDM have shown alterations in maternal hepatic electron transport chain and UCP2, a marker of ROS (38). Offspring of mice with GDM display impaired mitochondrial function and hepatic lipid accumulation (38, 39). The purpose of this study was to evaluate the impact of GDM on hepatic lipid metabolism, mitochondrial function, and inflammation in the maternal liver. It is imperative to understand how GDM and MASLD are connected. By evaluating the liver at varying time points during gestation, we can further understand the natural changes that occur in the liver in a healthy vs. GDM pregnancy. We hypothesized that GDM will significantly increase maternal hepatocellular injury.

## Materials and methods

### Experimental animals

The Baylor College of Medicine Institutional Animal Care and Use Committee approved all animal procedures. All methods performed were in accordance with the Guide for the Care and Use of Laboratory animals. Seven-week-old female and 12-week-old male wild type C57BL/6J were purchased from JAX (Bar Harbor, ME). Mice were kept at 23°C, 40-60% humidity, and a 14-hour light/10-hour dark cycle. Mice had ab libitum access to food and water. Female mice were mated to C57BL/6J breeder males for five days. Following, observation of a copulatory plug was identified and marked as day 0 of pregnancy. A total of 60 mice were included in this study. CD: day 0 n=8, day 6.5 n=8, day 13.5 n=4, and day 17.5 n=10. GDM: day 0 n=8, day 6.5 n=5, day 13.5 n=7, and day 17.5 n=10.

### Diet and timeline

Mice were randomized and fed either a high-fat, high-sucrose (HFHS) diet (D12451, Research Diets Inc., New Brunswick, NJ) or control diet (CD) (D12450K, Research Diets Inc.). Maternal feeding started 1-week prior to conception and continued throughout pregnancy. This feeding timeline has been verified to confirm a model of GDM in C57BL/6J mice (40–42). Livers were collected on days 0, 6.5, 13.5, and 17.5 of pregnancy. After a 6-hour fast, mice were euthanized via CO_2_ inhalation and cardiac exsanguination.

### Histology

Liver tissues fixed in 10% formalin for 24 hours were embedded in paraffin, sectioned, and stained with hematoxylin-eosin (H&E) by IDEXX BioAnalytics (Columbia, MO, USA) for histological evaluation. Steatosis was scored by direct observation of the percentage of overall surface area covered by lipid accumulation within cells on a low to medium power and scored as a 0 (<5%), 1 (5-33%), 2 (33-66%), or 3 (>66%). Lobular inflammation refers to the number of foci with white blood cell infiltrate present per 200x powered field, scored as 0 (no foci), 1 (<2 foci), 2 (2-4 foci), or 3 (>4 foci). Hepatocyte ballooning refers to the presence of hepatocytes that have pathologically swollen with or without the presence of lipid vacuoles, indicating a trend towards apoptosis and necrosis of the cell, and is scored as 0 (no ballooned hepatocytes), 1 (rare but definite ballooned cells), or 2 (many/most cells are prominent with ballooning). NAS (NAFLD Activity Score) assessment consists of histologically examining the liver for steatosis, lobular inflammation, and hepatocyte ballooning utilizing H&E staining, and is indicated as a sum of these scores (0–8) (43).

### Serum triglycerides and insulin

Triglycerides were isolated from the liver utilizing previous methods (44). Once extracted, liver and serum triglycerides were measured using the Serum Triglyceride Determination Ket (Sigma-Aldrich). Serum insulin was measured after a 6-hour fast using a Rat/Mouse Insulin ELISA kit from EMD Millipore according to the manufacturer’s instructions. Serum insulin and triglycerides were part of previous studies that have been published (40–42).

### Mitochondrial activity

β-hydroxyacyl-CoA dehydrogenase (β-HAD) and citrate synthase activities were measured utilizing methods from Srere et al. (45) and Bass et al. (46) as previously described by our lab (28, 47, 48). For citrate synthase, liver homogenates were incubated with acetyl-CoA, DTNB, and oxaloacetate. Following, detection of reduced DTNB at a wavelength of 412nm served as an index of enzymatic activity. For β-HAD, liver homogenate was placed in an assay buffer of EDTA, triethanolamine-HCl, and NADH at a pH of 7.0. After baseline reading, acetoacetyl-CoA was added, and the rates of NADH disappearance to NAD appearance ratio were measured every 10 seconds for 5 minutes at 340 nm.

### RNA extraction and quantitative PCR

Gene expression was completed from whole liver tissue samples. RNA and complementary DNA were isolated based on previous protocols reported from our lab (48, 49). Quantitative Real-time PCR (qPCR) was conducted using iTAQ Universal SYBR Green Supermix (Bio-Rad). Results are displayed as RQ (Relative Quantification) and was calculated using the Ct of the target gene, Ct of the housekeeping gene, and the average control group (day 0, CD). Results were calculated using the delta Ct methods and values are expressed relative to the control group (day 0, CD). Primer sequences are shown in **Supplementary Table 1**.

### Western blotting

Whole liver homogenates were prepared for Western blot analysis as previously described (28, 47, 49, 50). Primary antibodies were used at 1:1,000 dilutions, and secondary antibodies at 1:5,000 dilutions. Primary antibodies used are listed in **Supplementary Table 1**. Blots were analyzed via densiometric analysis (Image Lab v5.1, Bio-Rad Laboratories Inc., Hercules, CA). Total protein was assessed with Amido black (0.1%; Millipore Sigma) to control differences in protein loading and transfer, as previously described (28, 47, 49, 50).

### Statistical analysis

Statistical analyses were completed using GraphPad Prism 10.0.2. All data was analyzed via two-way ANOVA (diet, time) with Tukey’s multiple comparison *post-hoc* test employed when necessary. Differences were considered statistically significant when P < 0.05. Data are shown as mean ± SEM.

## Results

### Animal characteristics and MASLD development

**Figure 1:**
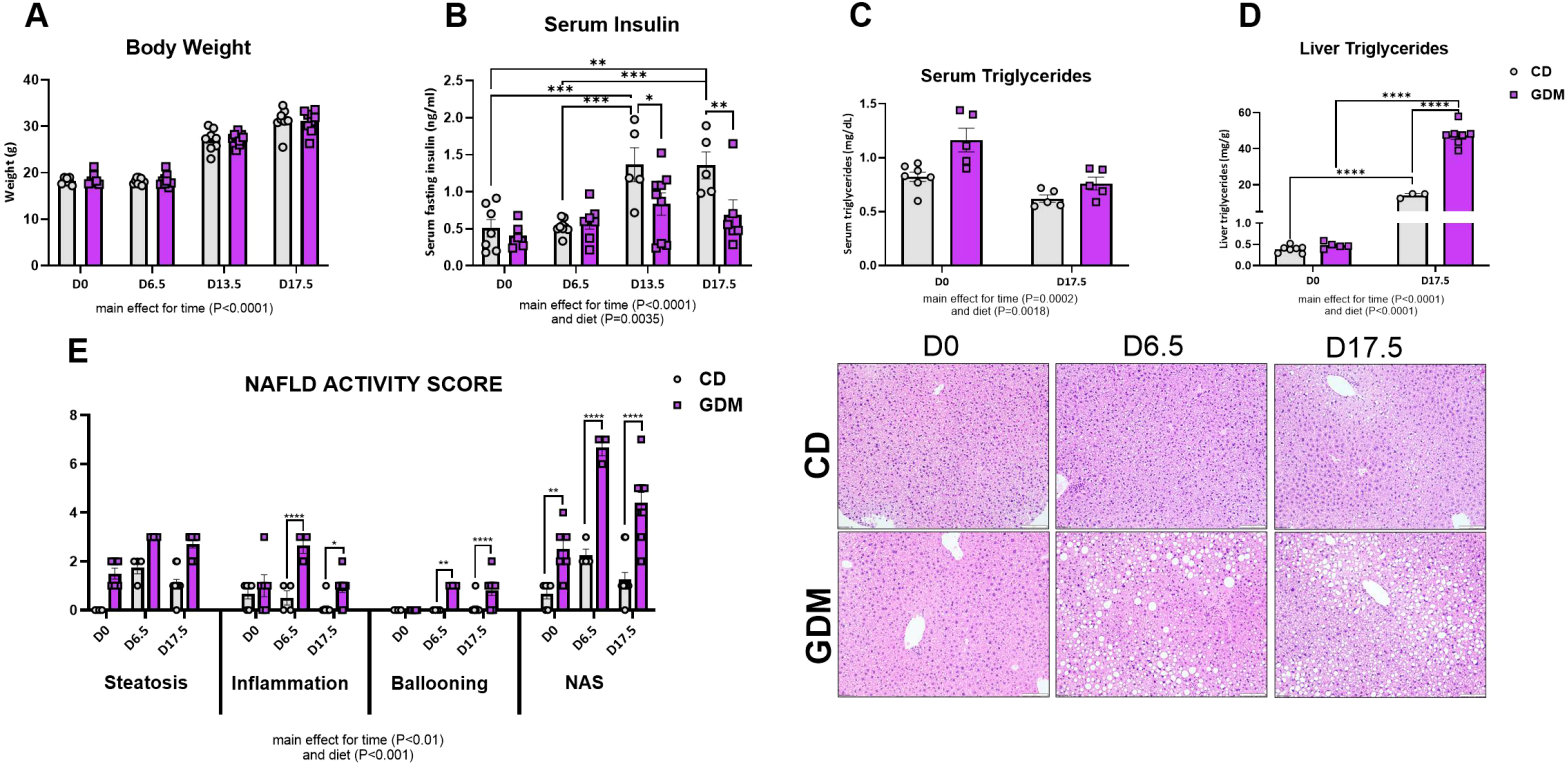
Animal characteristics. **(A)** Body weight of dams throughout stages of pregnancy (g). **(B)** Fasting serum insulin concentrations (ng/ml). **(C)** Serum triglycerides (mg/dL). Data were previously reported (40–42). **(D)** Liver triglycerides (mg/dL). **(E)** NAFLD Activity Score and Liver H&E Representative images. D0 = day 0, D6.5 = day 6.5, D13.5 = day 13.5, D17.5 = day 17.5 of pregnancy. *P<0.05, **P<0.01, ***P<0.001, ****P<0.0001 all denote an interaction. Main effect for time and/or diet indicated under each graph. Error bars represent SEM. N=6-10. CD, Control Diet; GDM, Gestational Diabetes Mellitus.

As we have previously reported for this mouse model of GDM, a significant increase in body weight across pregnancy stages occurred in both groups with a main effect for time (**Figure 1A**, P<0.0001). There were significant main effects for diet and time for serum insulin, serum triglycerides, and liver triglycerides (P<0.01). On days 13.5 and 17.5, serum insulin increased in CD, whereas GDM caused insulin secretion to be blunted (P=0.01, P=0.004, **Figure 1B**). This model, employed by our group, also exhibited systemic insulin resistance compared to control by day 13.5, as confirmed by glucose tolerance test and euglycemic-hyperinsulinemic clamp studies (41, 42). GDM mice compared to control mice displayed a non-significant elevation in serum triglycerides at day 0, but both groups were decreased by day 17.5 (**Figure 1C**). Liver triglycerides were markedly elevated in the GDM mice compared to control at day 17.5 (P<0.0001, **Figure 1D**). NAFLD activity score (NAS) and its three main components were evaluated to determine MASLD progression (**Figure 1E**). GDM displayed elevations in hepatic steatosis and significant increases in total NAS for day 0 (P<0.01), 6.5 (P<0.0001), and 17.5 (P<0.0001) compared to control mice. Hepatic inflammation and ballooning were also increased in GDM mice compared to CD at day 6.5 and 17.5 (P<0.05). There was a main effect for time and diet for all three components and NAS (P<0.01).

### Markers of hepatic lipid metabolism decreased throughout gestation

**Figure 2:**
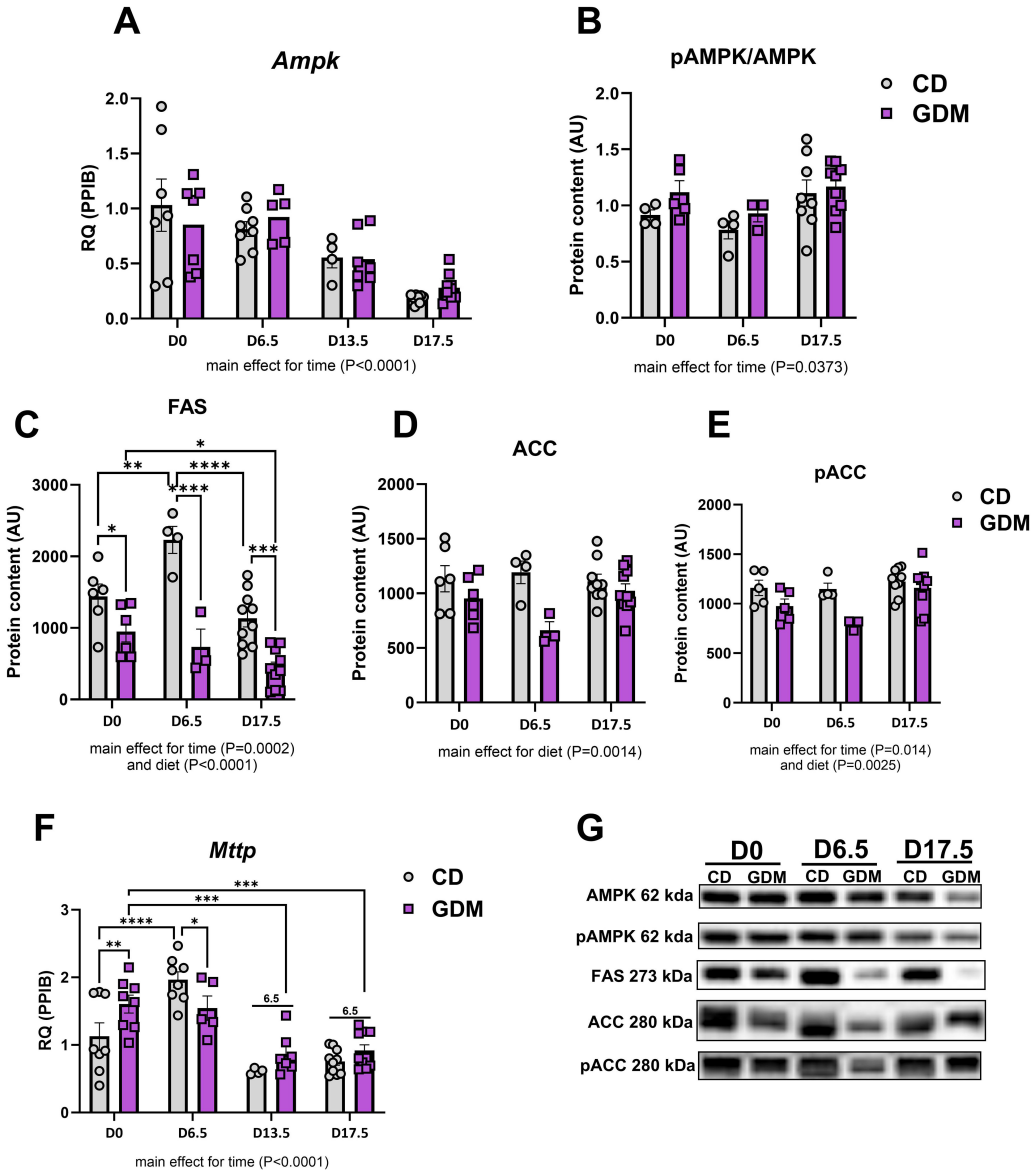
Hepatic lipid handling. **(A)** *Ampk* mRNA expression. Protein expression of: **(B)** pAMPK/AMPK, **(C)** FAS, **(D)** ACC, **(E)** pACC. **(F)** *Mttp* mRNA expression. **(G)** Representative protein bands from western blot for: AMPK, pAMPK, FAS, ACC, pACC. D0 = day 0, D6.5 = day 6.5, D13.5 = day 13.5, D17.5 = day 17.5 of pregnancy **(H)**. *P<0.05, **P<0.01, ***P<0.001, ****P<0.0001, all denote an interaction. 6.5: an interaction of both respective groups compared to day 6.5 with P<0.05. Main effect for time and/or diet indicated under each graph. Error bars represent SEM. N=8-10. CD, Control Diet; GDM, Gestational Diabetes Mellitus; RQ, Relative Quantification; AU, Arbitrary Units; PPIB, Peptidylprolyl Isomerase B, housekeeping gene.

Markers of hepatic lipid metabolism were evaluated throughout gestational development. mRNA expression of *Ampk* (AMP-activated protein kinase) decreased as pregnancy progressed (main effect for time, P<0.0001, **Figure 2A**) while protein expression of phospho-AMPK to AMPK decreased at day 6.5 but slightly increased by day 17.5 (main effect for time, P=0.0373, **Figure 2B**). Other major markers of lipid handling [fatty acid synthase (FAS), acetyl-CoA carboxylase (ACC), phospho-ACC, microsomal triglyceride transfer protein *(Mttp*)] except ACC (acetyl-CoA Carboxylase) protein were significantly decreased with gestational duration (P<0.05, **Figure 2**). GDM significantly decreased hepatic *de novo* lipogenesis marker FAS (fatty acid synthase) protein at day 0 (P=0.024), day 6.5 (P<0.0001), and 17.5 (P=0.0001) compared to CD (**Figure 2D**) and there were main effects for GDM for ACC and phospho-ACC protein content (P<0.01, **Figures 2E, F**). *Mttp*, which aids in transporting lipids across cell membranes, was elevated at day 0 (P=0.007) and then decreased at day 6.5 (P=0.035) in GDM compared to control mice (**Figure 2G**).

### Alterations in markers of mitochondrial turnover in GDM and gestation

**Figure 3:**
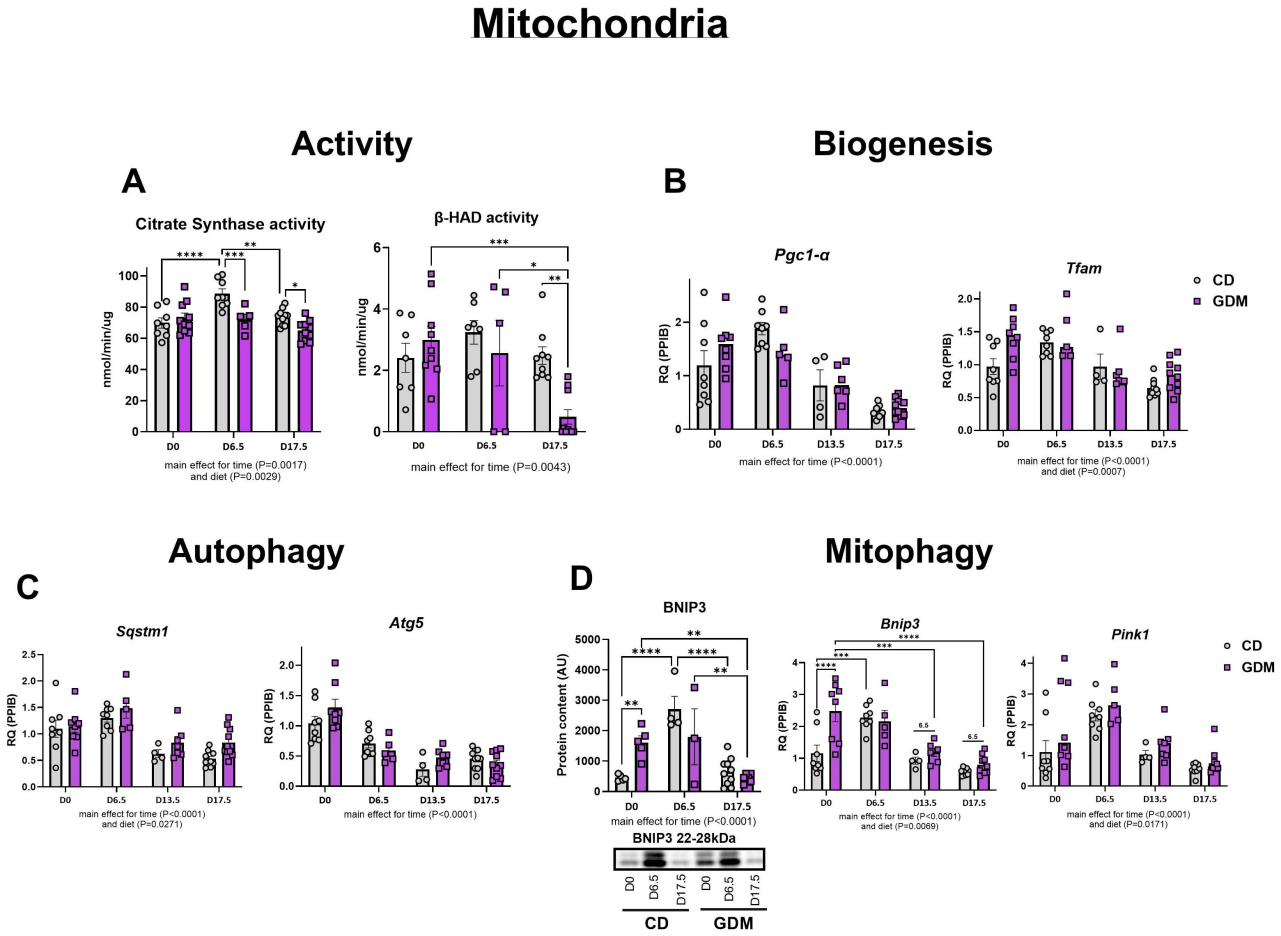
Hepatic mitochondrial activity, biogenesis, autophagy, and mitophagy. **(A)** Citrate synthase and (nmol/min/ug) and β-had activity (nmol/min/ug). **(B)** Biogenesis: mRNA expression of *Pgc1-α*, and *Tfam.* **(C)** Autophagy: mRNA expression of *Atg5* and *Sqstm1*. **(D)** Mitophagy: Protein expression of BNIP3 with its representative protein bands from western blot and mRNA expression of *Pink1* and *Bnip3.* D0 = day 0, D6.5 = day 6.5, D13.5 = day 13.5, D17.5 = day 17.5 of pregnancy. *P<0.05, **P<0.01, ***P<0.001, ****P<0.0001 all denote an interaction. 6.5: an interaction of both respective groups compared to day 6.5 with P<0.05. Main effect for time and/or diet indicated under each graph. Error bars represent SEM. N=4-10. CD, Control Diet; GDM, Gestational Diabetes Mellitus; RQ, Relative Quantification; AU, Arbitrary Units; PPIB, Peptidylprolyl Isomerase B, housekeeping gene.

Measures of hepatic mitochondrial function, citrate synthase and β-HAD activities, significantly decreased in dams with GDM at days 6.5 (P=0.001), 13.5 (P=0.019) and 17.5 (P=0.003) (**Figure 3A**). Markers of mitochondrial biogenesis, *Pgc1-α* (peroxisome proliferator-activated receptor gamma coactivator 1-alpha)*, and Tfam* (mitochondrial transcription factor A) exhibited parallel declines as pregnancy progresses (main effect of time, P<0.0001, **Figure 3B**). Markers of autophagy (*Atg5:* Autophagy-related protein 5, *Sqstm1*: Sequestosome-1) and mitophagy (*Pink1:* PTEN Induced Kinase *1, Bnip3:* BCL2 Interacting Protein 3) in both groups were significantly attenuated as gestation progressed to day 17.5 (main effect for time, P<0.0001, **Figures 3C, D**). GDM significantly increased BNIP3 protein and mRNA expression at day 0 compared with CD mice (P=0.004 and P<0.001, respectively, **Figure 3D**) but had similar expression to CD by day 17.5. Interestingly, there was a main effect for diet with GDM mice exhibiting elevated protein and/or mRNA markers of mitochondrial biogenesis (*Tfam*), autophagy (*Sqstm1*), mitophagy (BNIP3, *Bnip3, Pink1*) (P<0.05).

### GDM elevates markers of inflammation and antioxidant defense

**Figure 4:**
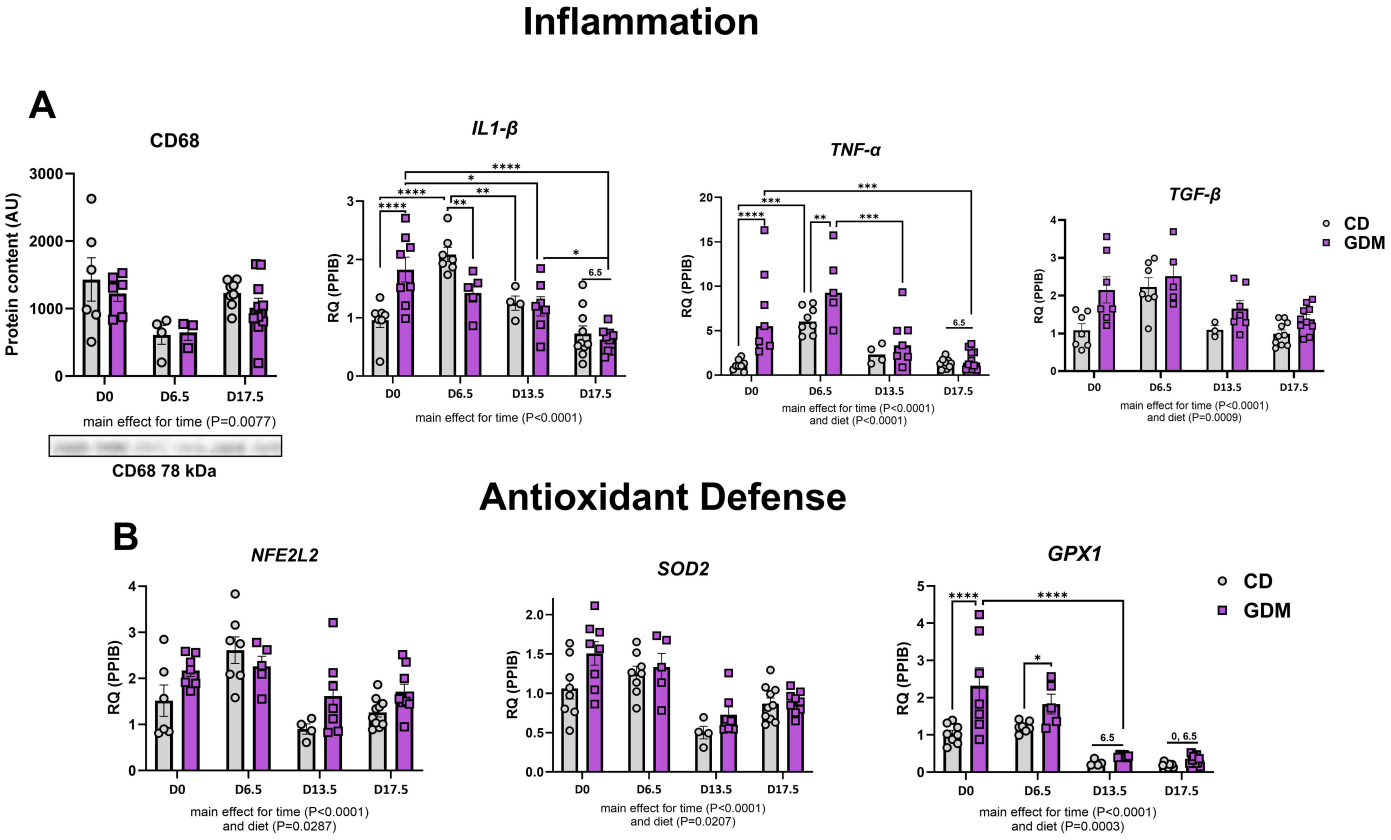
Markers of hepatic inflammation and antioxidant defense. **(A)** Inflammation: Protein expression of CD68 with its representative protein bands from western blot, and mRNA expression of *Il1-β*, *Tnf-α*, and Tgf-*β*. **(B)** Antioxidant defense: mRNA expression of *Nfe2l2*, *Sod2* and *Gpx1*. D0 = day 0, D6.5 = day 6.5, D13.5 = day 13.5, D17.5 = day 17.5 of pregnancy. *P<0.05, **P<0.01, ***P<0.001, ****P<0.0001 all denote an interaction. 6.5: an interaction of both respective groups compared to day 6.5 with P<0.05. 0: an interaction of both respective groups compared to day 0. Main effect for time and/or diet indicated under each graph. Error bars represent SEM. N=4-10. CD, Control Diet; GDM, Gestational Diabetes Mellitus; RQ, Relative Quantification; AU, Arbitrary Units; PPIB, Peptidylprolyl Isomerase B, housekeeping gene.

GDM had no effect on the protein expression of hepatic CD68 (cluster of differentiation 68), a marker of macrophage infiltration, but there was a main effect for time (P=0.0077, **Figure 4A**). Time during gestation also significantly decreased other markers of inflammation [*Il-1β* (interleukin-1 beta)*, Tnf-α* (tumor necrosis factor-alpha), and *Tgf-β* (transforming growth factor β)] and hepatic markers of antioxidant defense [*Nfe2l2 (*nuclear-factor erythroid-derived 2-like 2*), Sod2* (superoxide dismutase 2)*, Gpx1* (glutathione peroxidase 1)] (**Figure 4B**). GDM markedly upregulated *Il-1β*, *Tnf-α*, and Gpx1 at day 0 (P<0.0001, **Figure 4A**). There was also a main effect for diet with increases in hepatic Tnf-α (P<0.001), *Tgf-β* (P=0.0009), *Nfe2l* (P=0.0287), *Sod2* (P=0.0207), and *Gpx1* (P=0.0003) (**Figure 4B**).

## Discussion

Gestational diabetes mellitus is associated with an increased risk of MASLD, but there is a paucity of data mechanistically linking these two conditions. Here we provide novel evidence in an established high fat, high sucrose-fed mouse model of GDM (40–42) that changes in maternal liver lipid metabolism may play an important role in GDM pathology. GDM mice developed hepatic steatosis, the accumulation of lipid in the liver, with increases in hepatocellular inflammation and ballooning degeneration, consistent with MASH (35). The GDM mice also exhibited global downregulation in markers of lipid metabolism, and mitochondrial function, biogenesis, mitophagy, and autophagy with increasing gestation time.

Heightened insulin resistance is the distinguishing feature of GDM that is commonly accompanied by inadequate pancreatic beta cell expansion (51). Obesity is a risk factor for the development of GDM (and MASLD), but it has been found that up to one third of women with GDM are considered lean (BMI < 18.5 kg/m^2^) (52). To better characterize the condition, GDM is now being classified into subtypes dependent on insulin metabolism. Approximately 50% of women with GDM will have systemic insulin resistance, 35% will display insufficient insulin secretion, and 15% will exhibit variations of altered insulin metabolism (53–55). These data highlight the importance of understanding and investigating the varying pathophysiology of GDM. The mouse model used in the current study exhibited systemic insulin resistance and impaired insulin secretion with attenuated beta cell expansion, making it a preferred translational model to investigate GDM (40–42).

During healthy gestation, several metabolic adaptations occur such as elevated glucose utilization, decreases in fatty acid oxidation in the liver, and subsequent systemic insulin resistance (56, 57). As pregnancy progresses into its later stages, adipose tissue undergoes increased lipolysis to ensure sufficient circulating free fatty acids (58). GDM appears to extend these adaptations beyond what is metabolically necessary, aligning with MASLD development. Here, we found in our model of GDM that maternal liver triglycerides were significantly elevated at day 17.5 compared to control diet fed mice, while serum triglycerides were markedly decreased (**Figure 1**). Similarly, NAS, which includes steatosis, was markedly increased at day 0, 6.5, and 17.5 in GDM compared to CD with elevations occurring as gestation processed. Our data showed that markers of lipid breakdown (*Ampk*, pAMPK/AMPK) and export (*Mttp*) decreased as gestation progresses, in both groups. These data suggest that increased hepatic lipid uptake occurs in GDM, concomitant with significant decreases in lipid breakdown and export. This outcome was accompanied with decreased levels of FAS and ACC, markers of hepatic *de novo* lipogenesis (DNL), which is the formation of fats in the liver from other sources such as sugar (59). Decreased markers of hepatic DNL correspond with other short-term high fat diet feedings studies that show similar reductions (60–64). Overall, these data suggest GDM significantly decreases in lipid breakdown and export, eliciting the formation of lipid accumulation in the liver.

The mitochondrion is the main residence for lipid import and break down. The dysfunction of this organelle is an important feature of MASLD development (27, 28). With our findings of altered lipid storage and export, we hypothesized that our model of GDM would display significant alterations in hepatic mitochondrial function, aligning with MASLD progression. Citrate synthase and β-HAD activities, enzymes that catalyze the TCA cycle and fatty acid β-oxidation within the mitochondria, respectively, were both significantly decreased in GDM mice compared to control mice by day 17.5 (65). Interestingly, early in GDM pregnancy, markers of mitochondrial activity (citrate synthase, β-HAD), biogenesis (*Tfam*), autophagy (*Atg5*), and mitophagy (BNIP3, *Bnip3, Pink1*) were either significantly elevated or trending. Similar outcomes were seen with inflammation (*Il1-β, Tnf-α, Tgf-β*) and antioxidant defense (*Sod2, Gpx1*). This information suggests that early pregnancy is a critical window where transcriptional hepatic adaptations to GDM first emerge. In line with these findings, work from our group have previously shown that mitochondrial dysfunction actually precedes MASLD development (28), and findings from others suggest that alterations in plasma lipidomics in early pregnacy predict GDM development (21–23). It is known that pregnancy requires great metabolic flexibility, and substrate and additional energy requirements by the fetus, placenta, and uterus take priority (66–68). Regardless of GDM or control conditions, we found that all measures of mitochondrial turnover, lipid metabolism, inflammation, and antioxidant defense were attenuated by day 17.5 of pregnancy. Early pregnancy displays the largest alterations in hepatic mRNA and protein markers between GDM and CD, but it is not until later in pregnancy that the liver cannot compensate, and it develops overt metabolic characteristics of MASLD exhibited by increased liver triglycerides, elevated NAS (steatosis, inflammation, and ballooning), and attenuated mitochondrial activity.

One of the strengths of this study is the use of an established diet-fed mouse model to develop GDM. This model closely mimics key metabolic features of human GDM, such as insulin resistance and impaired insulin secretion. Furthermore, this is the first study to evaluate the impact of GDM on hepatic markers of mitochondrial activity, biogenesis, mitophagy, autophagy, inflammation, antioxidant defense, and lipid metabolism throughout gestation. Despite these strengths, there are limitations. While the HFHS-fed mouse model does recapitulate GDM pathophysiology, it is important to note that pregnancy in a human is much lengthier and likely more complex, limiting the translation of these findings from rodent GDM to human GDM. Secondly, the present study does not include any non-pregnant mice as a control group. Although previous literature suggests that control-diet fed non-pregnant mice would not display any hepatic metabolic alterations over time (69–72), direct comparisons with this experimental setting would help strengthen the conclusions.

In conclusion, our data demonstrates that HFHS feeding in a GDM model leads to increased maternal hepatic triglyceride accumulation, elevated NAS, and reduced mitochondrial activity, accompanied by increases in hepatic inflammation and antioxidant defense. Pregnancy itself acts as a powerful metabolic switch, reprogramming nutrient utilization and energy balance to support fetal development. This metabolic shift appears strong enough to override liver adaptations to high-fat diet-induced GDM, ensuring continued development of the fetus and support organs but leading to hepatic lipid increases. To our knowledge, this is the first study to evaluate and characterize alterations in hepatic mitochondrial and lipid metabolism during both normal gestation and in a GDM model. These findings highlight the significant impact of gestation and GDM on liver health and MASLD risk.

## Data Availability

The original contributions presented in the study are included in the article/**Supplementary Material**. Further inquiries can be directed to the corresponding authors.

## Glossary

GDM: Gestational Diabetes Mellitus
MASLD: Metabolic Dysfunction-Associated Steatotic Liver Disease
MASH: Metabolic-Dysfunction Associated Steatohepatitis
CD68: Cluster of Differentiation 68
ACC: Acetyl-CoA Carboxylase
pACC: phosphorylated Acetyl-CoA Carboxylase
FAS: Fatty acid synthase
TGF-β: Transforming growth factor beta
NFE2L2/NRF2: Nuclear factor, erythroid 2-like 2
β-HAD: β-hydroxyacyl-CoA dehydrogenase
AMPK: AMP-activated protein kinase
PPIB: Peptidylprolyl Isomerase B
PGC1-α: Peroxisome proliferator-activated receptor gamma coactivator 1-alpha
TFAM: Mitochondrial transcription factor A
PINK1: PTEN Induced Kinase 1
SQSTM1: Sequestosome 1
BNIP3: BCL2 interacting protein 3
ATG5: Autophagy protein 5
SOD2: Superoxide dismutase 2
GPX1: Glutathione peroxidase 1
TNF-α: Tumor necrosis factor alpha
IL1-β: Interleukin-1 beta
MTTP: Microsomal triglyceride transfer protein
