# Supplementary material for: A murine model of gestational diabetes reveals MASLD risk and alterations in markers of hepatic mitochondrial metabolism
Source: Front Endocrinol (Lausanne). 2025 Jun 25;16:1498764. doi: 10.3389/fendo.2025.1498764 (PMC12237674; doi:10.3389/fendo.2025.1498764)
Supplement: Supplementary file 1 [file Table1.docx]

**Western blotting primary antibodies**

The primary antibodies were used at followed: AMP-activated Protein Kinase alpha (AMPKα; #2532S, Cell signaling Technologies), Phosphorylated AMP-activate Protein Kinase alpha Thr172 (pAMPKα; #2531, Cell signaling Technologies), Fatty Acid Synthase (FAS; #3189, Cell signaling Technologies), Acetyl-coA Carboxylase (ACC; #3662, Cell Signaling), phosphorylated Acetyl-coA Carboxylase (pACC; #3661, Cell signaling Technologies), BCL2 Interacting Protein 3 (BNIP3; #3769S, Cell Signaling Technologies), Cluster of Differentiation 68 (CD68; #20060, Santa Cruz Biotechnology).

| **Primers** | **Forward Sequence 5’-3’** | **Reverse Sequence 5’-3’** |
| --- | --- | --- |
| *PPIB* | TGGAGATGAATCTGTAGGAC | CAAATCCTTTCTCTCCTGTAG |
| *PGC1-α* | AGTGGTGTAGCGACCAATCG | TCTTCATCCACGGGGAGACT |
| *ATG5* | CACCCTGAAATGAGTTTCCAG | AAAGTGAGCCTCAACCGCAT |
| *SQSTM1/P62* | AATGTGATCTGTGATGGTTG | GAGAGAAGCTATCAGAGAGG |
| *PINK1* | GTGGGACTCAGATGGCTGTC | TCTACACTGGAGCTGTTGAAAGG |
| *BNIP3* | ACCACAAGATACCAACAGAG | AATCTTCCTCAGACAGAGTG |
| *TFAM* | ACAAGCTTCAATTTTCCCTG | GACCTCGTTCAGCATATAAC |
| *AMPK* | CCGGGAAAGGAGCACAAGAT | GGAGCTTGGAGTCGGGC |
| *SOD2* | AGGAGAGTTGCTGGAAGGCTA | TAGTAAGCGTGCTCCCACAC |
| *GXP1* | GGAGAATGGCAAGAATGAAG | TTCGCACTTCTCAAACAATG |
| *TNF-α* | AGGCATCCCCCAAAAGATG | CTTGGTGGTTTGCTACGACG |
| *IL1-β* | TCACAGCAGCACATCAACAA | TGTCCTTCATCCTGGAAGTC |
| *MTTP* | CAAAGGAACAGAGCTTCATGGT | ATAAGCCCTTTCACCACGCT |
| *TGF-β* | AAGTTGGCATGGCCTT | GCCCTGGATACCACCTATTGC |
| *NFE2L2* | GTGGAGACTTAACCAAAGAG | GATGACTGTCAAAAACCTC |
